# Impaired kidney function is associated with lower quality of life among community-dwelling older adults: The screening for CKD among older people across Europe (SCOPE) study

**DOI:** 10.1186/s12877-020-01697-3

**Published:** 2020-10-02

**Authors:** Rada Artzi-Medvedik, Robert Kob, Paolo Fabbietti, Fabrizia Lattanzio, Andrea Corsonello, Yehudit Melzer, Regina Roller-Wirnsberger, Gerhard Wirnsberger, Francesco Mattace-Raso, Lisanne Tap, Pedro Gil, Sara Lainez Martinez, Francesc Formiga, Rafael Moreno-González, Tomasz Kostka, Agnieszka Guligowska, Johan Ärnlöv, Axel C. Carlsson, Ellen Freiberger, Itshak Melzer, on behalf of the SCOPE investigators, Fabrizia Lattanzio, Silvia Bustacchini, Silvia Bolognini, Paola D’Ascoli, Raffaella Moresi, Giuseppina Di Stefano, Cinzia Giammarchi, Anna Rita Bonfigli, Roberta Galeazzi, Federica Lenci, Stefano Della Bella, Enrico Bordoni, Mauro Provinciali, Robertina Giacconi, Cinzia Giuli, Demetrio Postacchini, Sabrina Garasto, Annalisa Cozza, Francesco Guarasci, Sonia D’Alia, Romano Firmani, Moreno Nacciariti, Mirko Di Rosa, Paolo Fabbietti

**Affiliations:** 1 Department of Nursing, Recanati School for Community Health Professions at the faculty of Health Sciences, Ben-Gurion University of the Negev, Beer-sheva, Israel; 2 Department of Physical Therapy, Recanati School for Community Health Professions at the faculty of Health Sciences, Ben-Gurion University of the Negev, Beer-sheva, Israel; 3 Department of Internal Medicine-Geriatrics, Institute for Biomedicine of Aging, Krankenhaus Barmherzige Brüder, Friedrich-Alexander Universität Erlangen-Nürnberg, Koberger Strasse 60, 90408 Nuremberg, Germany; 4 Italian National Research Center on Aging (IRCCS INRCA), Ancona, Fermo and Cosenza Italy; 5 Laboratory of Geriatric Pharmacoepidemiology and Biostatistics, IRCCS INRCA, Via S. Margherita 5, 60124 Ancona, Italy; 6 Maccabi Health Organization, Negev district, Tel Aviv-Yafo, Israel; 7 Department of Internal Medicine, Medical University of Graz, Graz, Austria; 8 Division of Nephrology, Department of Internal Medicine, Medical University of Graz, Graz, Austria; 9 Department of Internal Medicine, Section of Geriatric Medicine, Erasmus MC, University Medical Center Rotterdam, Rotterdam, The Netherlands; 10 Department of Geriatric Medicine, Hospital Clinico San Carlos, Madrid, Spain; 11 Geriatric Unit, Internal Medicine Department, Bellvitge University Hospital – IDIBELL – L’Hospitalet de Llobregat, Barcelona, Spain; 12 Department of Geriatrics, Healthy Ageing Research Centre, Medical University of Lodz, Lodz, Poland; 13 Department of Medical Sciences, Uppsala University, Uppsala, Sweden; 14 School of Health and Social Studies, Dalarna University, Falun, Sweden; 15 Division of Family Medicine, Department of Neurobiology, Care Sciences and Society, Karolinska Institutet, Huddinge, Sweden

**Keywords:** Quality of life, Chronic kidney disease, Old adults

## Abstract

**Background:**

Quality of life (QoL) refers to the physical, psychological, social and medical aspects of life that are influenced by health status and function. The purpose of this study was to measure the self-perceived health status among the elderly population across Europe in different stages of Chronic Kidney Disease (CKD).

**Methods:**

Our series consisted of 2255 community-dwelling older adults enrolled in the Screening for Chronic Kidney Disease (CKD) among Older People across Europe (SCOPE) study. All patients underwent a comprehensive geriatric assessment (CGA), including included demographics, clinical and physical assessment, number of medications taken, family arrangement, Geriatric Depression Scale (GDS), Cumulative Illness Rating Scale, History of falls, Lower urinary tract symptoms, and Short Physical Performance Battery (SPPB). Estimated glomerular filtration rate (eGFR) was calculated by Berlin Initiative Study (BIS) equation. Quality of life was assessed by Euro Qol questionnaire (Euro-Qol 5D) and EQ-Visual Analogue Scale (EQ-VAS). The association between CKD (eGFR < 60, < 45 ml or < 30 ml/min/1.73m^2^) and low EQoL-VAS was investigated by multivariable logistic regression models.

**Results:**

CKD was found to be significantly associated with low EQoL-VAS in crude analysis (OR = 1.47, 95%CI = 1.16–1.85 for eGFR< 60; OR = 1.38, 95%CI = 1.08–1.77 for eGFR< 45; OR = 1.57, 95%CI = 1.01–2.44). Such association was no longer significant only when adjusting for SPPB (OR = 1.20, 95%CI = 0.93–1.56 for eGFR< 60; OR = 0.87, 95%CI = 0.64–1.18 for eGFR< 45; OR = 0.84, 95%CI = 0.50–1.42), CIRS and polypharmacy (OR = 1.16, 95%CI = 0.90–1.50 for eGFR< 60; OR = 0.86, 95%CI = 0.64–1.16 for eGFR< 45; OR = 1.11, 95%CI = 0.69–1.80) or diabetes, hypertension and chronic obstructive pulmonary disease (OR = 1.28, 95%CI = 0.99–1.64 for eGFR< 60; OR = 1.16, 95%CI = 0.88–1.52 for eGFR< 45; OR = 1.47, 95%CI = 0.92–2.34). The association between CKD and low EQoL-VAS was confirmed in all remaining multivariable models.

**Conclusions:**

CKD may significantly affect QoL in community-dwelling older adults. Physical performance, polypharmacy, diabetes, hypertension and COPD may affect such association, which suggests that the impact of CKD on QoL is likely multifactorial and partly mediated by co-occurrent conditions/risk factors.

## Background

The importance of Quality of life (QoL) in old age was acknowledged in the WHO report on healthy aging 2015 [1]. However, rising life expectancy worldwide is not limited to the healthy population, but also affects subpopulations with a history of disease, which contribute to make QoL a relevant outcome in terms of public health among older people [2].

QoL is basically a subjective condition that expresses how people are satisfied with their life and the degree of wellbeing and happiness they feel [3]. Health-related QoL refers to the physical, psychological, social, spiritual aspects of QoL that are influenced by health and health-related events such as diseases and their treatments [4, 5].

Chronic kidney disease (CKD) is among chronic diseases significantly affecting QoL among older people. Besides exerting a major effect on global health, either as a risk factor for morbidity and mortality or by causing cardiovascular disease [6], the burden of CKD in older people is also related to its complications, including impaired physical function [7, 8], frailty [9, 10], cognitive impairment [11], vision impairment [12], malnutrition [13], and sarcopenia [14]. All the above may influence QoL of older adults.

Most studies showed that severe CKD and dialysis have negative impact on Health Related QoL [15, 16]. However, due to the slow and unpredictable nature of CKD trajectories, earlier CKD stages (e.g. stage 3a and 3b) may also significantly affect Health Related QoL [17]. The few studies investigating the impact of early stages of CKD on QoL show that QoL may be poorer than that of the general population, but better than for CKD patients on dialysis [18, 19]. In a recent systematic review, Yapa et al. [20] showed that health-related QoL may worsen when CKD symptoms (e.g. fatigue, exhaustion and drowsiness) appear.

The objective of this cross-sectional study was to investigate the QoL among older adults across Europe in early stages of CKD, in order to identify factors potentially influencing the relationship between kidney function and QoL.

## Methods

### Study design and participants

The SCOPE study (European Grant Agreement no. 436849), is a multicenter prospective cohort study involving patients older than 75 years attending geriatric and nephrology outpatient services in participating institutions in Austria, Germany, Israel, Italy, the Netherlands, Poland and Spain. Only people aged 75 or more were asked to participate because of the high prevalence of CKD in this population [21, 22]. Methods of the SCOPE study have been extensively described elsewhere [23]. Briefly, all patients attending the outpatient services at participating centers from August 2016 to August 2018 were asked to participate. Only patients signing a written informed consent entered the study. Age greater or equal to 75 years was the only inclusion criteria, the exclusion criteria were: end-stage renal disease or dialysis at time of enrollment; history of solid organ or bone marrow transplantation; active malignancy within 24 months prior to screening or metastatic cancer; life expectancy less than 6 months (based on the judgment of the study physician after careful medical history collection and diagnoses emerging from examination of clinical documentation exhibited); severe cognitive impairment (Mini Mental State Examination < 10); any medical or other reason (e.g. known or suspected patients’ inability to comply with the protocol procedure) in the judgement of the investigators, that the patient was unsuitable for the study; unwilling to provide consent and limited possibility to attend follow-up visits. Enrolled patients underwent an extensive assessment including: demographic data, socioeconomic status, physical examination, comprehensive geriatric assessment, bioimpedance analysis, diagnoses (clinical history and assessment of clinical documentation exhibited by patients and/or caregivers), quality of life, physical performance, overall comorbidity and blood and urine sampling. Patients were followed-up for 24-months as previously described [23]. The study protocol was approved by ethics committees at all participating institutions, and complies with the Declaration of Helsinki and Good Clinical Practice Guidelines. The study was registered at ClinicalTrials.gov (NCT02691546). Only baseline data was used in the present study.

Overall, 2461 patients were initially enrolled in the study; 206 patients were excluded because of incomplete baseline data, thus leaving a final sample of 2255 participants to be included in the present analysis.

### Study variables

QoL was assessed by EQ-Visual Analogue Scale (EQoL-VAS), that is part of the Euro-Quality of Life 5D (Euro-Qol 5D) [24–26]. The EQ-VAS asks participants to indicate their overall health on a vertical visual analogue scale, ranging from 0 “worst possible” to 100 “best possible” health. The Euro-Qol 5D is a standardized instrument for measuring generic health rated QoL measure with one question on five different dimensions that include mobility, self-care, usual activities, pain/discomfort, and anxiety/depression. The answers given to Euro-QoL 5D are scored from 1 “I have no problems … “ for perfect health to 5 “I am unable to …. “ for bad health status. The 5-digit numbers for the five dimensions are combined and describe the patient’s health state. The Euro-Qol 5D and EQoL-VAS was formerly validated in several different settings and clinical conditions [27–30].

Estimated glomerular filtration rate (eGFR) was calculated by Berlin Initiative Study (BIS) equation [31], and categorized as < 60, < 45 or < 30 ml/min/1.73m^2^.

Other variables included in the present study were: demographics, body mass index (BMI), number of diseases and medications taken, family arrangements; Basic (ADL) and Instrumental Activities of Daily Living (IADL) [32, 33]; Mini Mental State Exam (MMSE) [34]; 15-items Geriatric Depression Scale, GDS [35]; Cumulative Illness Rating Scale, CIRS [36]; History of falls; Lower urinary tract symptoms, LUTS [37]; hand grip strength [38]; Short Physical Performance Battery, SPPB [39]. Selected diagnoses, including diabetes, hypertension, stroke, hip fractures, chronic obstructive pulmonary disease, osteoporosis, Parkinson’s disease and anemia were also considered as potential confounders.

### Statistical analysis

**Descriptive analyses** of patients grouped according to EQoL-VAS (low EQoL-VAS, 0–50, intermediate EQoL-VAS, 51–75, and high EQoL-VAS, 75–100) were presented. The chi-square test was used for categorical variables and ANOVA one-way test for continuous ones. Post-hoc analysis for multiple comparisons was carried out by Bonferroni correction for continuous variables and by Dunn’s test for categorical ones. Therefore, multivariable logistic regression models were built to investigate the association between CKD (eGFR < 60, < 45 or < 30 ml/min/1.73m^2^) and low EQoL-VAS. Logistic regression models were as follows: crude (model 1), adjusted for age and gender (model 2), furtherly adjusted by adding family arrangement (i.e., being widow) (model 3), SPPB (model 4), falls (model 5); mood status i.e., GDS > 5 (model 6), Cumulative Illness Rating Score and number of medications≥5 (model 7), LUTS (model 8), comorbidities (models 9 and 10), and anemia (model 11). All statistical analyses were performed with SPSS statistical software package version 24 (SPSS Inc., Chicago, IL, USA). Statistical significance was set at *P* <  0.05.

## Results

Table 1 shows that 984 out of 2255 participants (43.6%) reported high EQoL-VAS, and 487 (21.6%) reported low EQoL-VAS. More than half (55.96%) were married or lived with a partner, 33.7% were widowed, 5.4% were single and 24.4% lived alone. Older adults with low EQoL-VAS (0–50) were more frequently women, single, and widowed, and had lower education compared to those with intermediate and with high EQoL-VAS (Table 1).

**Table 1:** Sociodemographic characteristics, according to EQoL-VAS category

Variable	All participants (***n*** = 2255)	Group A Low EQoL-VAS 0–50 (***n*** = 487)	Group B Intermediate EQoL-VAS 51–75 (***n*** = 784)	Group C High EQoL-VAS 76–100 (***n*** = 984)	***p-value***	post hoc
Euro QoL questionnaire (Euro-Qol 5D)	7.0 (4.0)	10.0 (5.0)	8.0 (4.0)	6.0 (3.0)	*< 0.001*	*a* vs. *b*
						*a* vs. *c*
						*b* vs. *c*
Age (years)	79.5 (5.9)	80.0 (6.4)	79.5 (6.0)	79.2 (5.3)	*0.009*	*a* vs. *c*
Sex: female	1255 (55.7)	332 (68.2)	423 (54.0)	500 (50.8)	*< 0.001*	*a* vs. *b*
						*a* vs. *c*
						*b* vs. *c*
Body mass index, BMI (kg/m^2^)	27.3 (5.7)	28.3 (6.1)	27.6 (5.9)	26.7 (5.3)	*< 0.001*	*a* vs. *c*
						*b* vs. *c*
**Marital status**						
Single	121 (5.4)	35 (7.2)	40 (5.1)	46 (4.7)	*< 0.001*	*a* vs. *b*
Married/ living with a partner	1253 (55.6)	227(46.6)	439(56.0)	587 (59.7)		*a* vs. *c*
Separated/divorced	120 (5.3)	31 (6.4)	47 (6.0)	42 (4.3)		
Widowed	761 (33.7)	194 (39.8)	258 (32.9)	309 (31.4)		
Education (years)	11.0 (7.0)	10.0 (5.0)	12.0 (7.0)	12.0 (7.0)	*< 0.001*	*a* vs. *b*
						*a* vs. *c*

NOTE. Values are mean ± SD for continuous normal distributions, n (%) for categorical variables, and median (interquartile range) for not normal distributions

Table 2 shows that eGFR was lower and the prevalence of CKD was higher among patients with low EQoL-VAS, whatever was the eGFR threshold used. Polypharmacy was also highly prevalent among patients with low EQoL-VAS, who also exhibited higher average CIRS score, greater prevalence of (LUTS) and comorbidities and lower hemoglobin values (Table 2).

**Table 2:** Clinical (**Medical conditions**) and laboratory parameters according to the EQoL-VAS category presented as N (%)

Variable	All participants (n = 2255)	Group A Low EQoL-VAS (0–50) (n = 487)	Group B Intermediate EQoL-VAS (51–75) (n = 784)	Group C High EQoL-VAS (76–100) (n = 984)	***p-value***	post hoc
**eGFR, ml/min/1.73 m**^**2**^	54.2 (19.6)	53.2 (19.7)	54.1 (21.2)	55.7 (18.4)	*0.005*	*a* vs. *c*
< 60	1423 (63.1)	336 (69.0)	494 (63.0)	593 (60.3)	*0.005*	*a* vs. *b*
						*a* vs. *c*
						*b* vs. *c*
< 45	560 (24.8)	137 (28.1)	206 (26.3)	217 (22.1)	*0.020*	*a* vs. *c*
						*b* vs. *c*
< 30	141 (6.3)	37 (7.6)	55 (7.0)	49 (5.0)	*0.082*	
Diabetes	568 (25.2)	137 (28.1)	225 (28.7)	206 (21)	*< 0.001*	*a* vs. *b*
						*a* vs. *c*
						*b* vs. *c*
Hypertension	1732 (76.8)	409 (84.0)	625 (79.7)	698 (70.9)	*< 0.001*	*a* vs. *b*
						*a* vs. *c b* vs. *c*
Stroke	131 (5.8)	44 (9.0)	50 (6.4)	37 (3.8)	*< 0.001*	*a* vs. *b*
						*a* vs. *c b* vs. *c*
Hip Fractures	111 (4.9)	36 (7.4)	44 (5.6)	31 (3.2)	*0.001*	*a* vs. *b*
						*a* vs. *c b* vs. *c*
Chronic obstructive pulmonary disease (COPD)	267 (11.8)	76 (15.6)	99 (12.6)	92 (9.3)	*0.002*	*a* vs. *b*
						*a* vs. *c*
						*b* vs. *c*
Osteoporosis	688 (30.5)	186 (38.2)	252 (32.1)	250 (25.4)	*< 0.001*	*a* vs. *b*
						*a* vs. *c*
						*b* vs. *c*
Parkinson’s disease	45 (2.0)	22 (4.5)	11 (1.4)	12 (1.2)	*< 0.001*	*a* vs. *b*
						*a* vs. *c*
Anemia	477 (21.2)	137 (28.1)	156 (19.9)	184 (18.7)	*< 0.001*	*a* vs. *b*
						*a* vs. *c*
Cumulative Illness Rating Score (CIRS)	8.0 (6.0)	9.0 (7.0)	8.0 (7.0)	7.0 (6.0)	*< 0.001*	*a* vs. *b*
						*a* vs. *c b* vs. *c*
Take ≥5 current medications	1509 (66.9)	389 (79.9)	554 (70.7)	566 (57.5)	*< 0.001*	*a* vs. *b*
						*a* vs. *c b* vs. *c*
Lower urinary tract symptoms (LUTS)	653 (29.0)	173 (35.5)	257 (32.8)	223 (22.7)	*< 0.001*	*a* vs. *b*
						*a* vs. *c*
						*b* vs. *c*
Hemoglobin (Hb)	13.5 ± 1.9	13.1 ± 1.5	13.5 ± 1.4	13.7 ± 1.4	*< 0.001*	*a* vs. *c*

NOTE. Values are mean ± SD for continuous normal distributions, n (%) for categorical variables, and median (interquartile range) for not normal distributions

In regard to physical and emotional status, SPPB scores and hand grip strength were lower and the prevalence of ADL/IADL dependency depression, cognitive impairment and history of falls was higher (Table 3).

**Table 3:** Physical, cognitive and emotional status according to the EQoL-VAS category

Variable	All participants (n = 2255)	Group A Low EQoL-VAS 0–50 (n = 487)	Group B Intermediate EQoL-VAS 51–75 (n = 784)	Group C High EQoL-VAS 76–100 (n = 984)	***p-value***	post hoc
ADL dependent or intensive assistance > = 1	107 (4.8)	50 (10.3)	33 (4.2)	24 (2.4)	*< 0.001*	*a* vs. *b*
						*a* vs. *c*
						*b* vs *c*
IADL dependent or intensive assistance > = 1	993 (44.1)	251 (51.9)	329 (42.0)	413 (42.0)	*< 0.001*	*a* vs. *b*
						*a* vs. *c*
						*b* vs. *c*
**SPPB total score (avarge ± SD)**	9 (4)	7 (6)	9 (4)	10 (3)	*< 0.001*	*a* vs *b*
						*a* vs *c*
**SPPB Balance score**						
*Held SBS < 10 s and held 10 s SBS but unable ST as severe balance limitation*	432 (20.1)	151 (34.0)	148 (20.0)	133 (13.8)	*< 0.001*	*a* vs. *b*
*Moderate limitation Held ST for 10 s und held FT till 9 s.*	410 (19.1)	99 (22.3)	143 (19.3)	168 (17.4)		
*No limitation: hold FT for 10 s.*	1307 (60.8)	194 (43.7)	450 (60.7)	663 (68.8)		*a* vs. *c*
						*b* vs. *c*
**SPPB gait score**						
*< 4.82 s*	1138 (50.5)	157 (32.2)	372 (47.4)	609 (61.9)	*< 0.001*	*a* vs. *b*
*4.82–8.7 s*	902 (40.0)	224 (46.0)	348 (44.4)	330 (33.5)		
						*a* vs. *c*
*> 8.70 s and Unable*	215 (9.5)	106 (21.8)	64 (8.2)	45 (4.6)		
						*b* vs. *c*
**5-sit to stand score**						
*≤ 11.19 s*	654 (32.2)	62 (16.5)	222 (31.2)	370 (39.2)	*< 0.001*	*a* vs. *b*
*11.20–16.69 s*	924 (45.5)	163 (43.4)	318 (44.7)	443 (46.9)		
						*a* vs. *c*
*> 16.70 s and > 60 s or unable*	454 (22.3)	151 (40.2)	171 (24.1)	132 (14.0)		
						*b* vs. *c*
Hand grip strength (avarge ± SD)	21.0 (12.8)	19.4 (8.3)	22.9 (9.3)	24.1 (9.2)	*< 0.001*	*a* vs. *b*
						*a* vs. *c*
						*b* vs. *c*
GDS score (avarge ± SD)	2 (3)	4 (4)	2 (3)	1 (3)	*< 0.001*	*a* vs. *b*
						*a* vs. *c*
GDS > 5	316 (14.0)	152 (31.2)	104 (13.3)	60 (6.1)	*< 0.001*	*a* vs. *b*
						*a* vs. *c*
						*b* vs. *c*
MMSE score (avarge ± SD)	29 (3)	28 (4)	29 (3)	29 (3)	*< 0.001*	*a* vs. *b*
						*a* vs. *c*
MMSE < 24	159 (7.1)	50 (10.3)	46 (5.9)	63 (6.4)	*0.007*	*a* vs. *b*
						*a* vs. *c*
						*b* vs. *c*
Fall at the past 12 months	746 (33.1)	207 (42.5)	267 (34.1)	272 (27.6)	*< 0.001*	*a* vs. *b*
						*a* vs. *c*
						*b* vs. *c*

NOTE. Values are mean ± SD for continuous normal distributions, n (%) for categorical variables, and median (interquartile range) for not normal distributions

**Abbreviations**: NS, not significance; ADL, activities of daily living; GDS, Geriatric Depression Scale; iADL, instrumental activities of daily living; MMSE, Mini-Mental State Examination; SPPB, Short Physical performance Battery

In logistic regression analyses (Table 4), CKD was significantly associated with the outcome independent of the eGFR threshold considered in the analysis. After adjusting for age, sex, being widowed, history of falls, GDS > 5, LUTS, stroke, hip fracture, Parkinson’s disease, and anemia the association between eGFR and low EQoL-VAS remained substantially unchanged (Table 4). When we adjusted for SPPB (model 4), the association between eGFR and the outcome was no longer significant. Indeed, SPPB score qualified as a significant negative correlate of low EQoL-VAS (OR = 0.72; 95%CI = 0.69–0.76 in the eGFR< 60 analysis, OR = 0.72; 95%CI = 0.68–0.75 in the GFR < 45 analysis and OR = 0.72; 95%CI = 0.68–0.75 in the GFR < 30 analysis). Similarly, CIRS (OR = 1.10; 95%CI = 1.07–1.13 in the eGFR< 60 analysis, OR = 1.11; 95%CI = 1.01–1.14 in the GFR < 45 analysis and OR = 1.10; 95%CI = 1.07–1.14 in the GFR < 30 analysis) and number of medications (OR = 1.98; 95%CI = 1.49–2.62 for eGFR< 60 analysis, OR = 2.01; 95%CI = 1.51–2.66 in the GFR < 45 analysis and OR = 1.99; 95%CI = 1.50–2.64 respectively in the GFR < 30 analysis) were significantly associated with the study outcome in model 7. Diabetes (OR = 1.42; 95%CI = 1.09–1.85 in eGFR< 60 analysis, OR = 1.40; 95%CI = 1.07–1.84 in eGFR< 45 analysis and OR = 1.42; 95%CI = 1.09–1.86 in eGFR< 30 analysis), hypertension (OR = 1.83; 95%CI = 1.36–2.45 in eGFR< 60 analysis, OR = 1.87; 95%CI = 1.40–2.51 in eGFR< 45 analysis and OR = 1.86; 95%CI = 1.39–2.49 in eGFR< 30 analysis), and chronic obstructive pulmonary disease (OR = 1.99; 95%CI = 1.41–2.82 in eGFR< 60 analysis, OR = 2.01; 95%CI = 1.43–2.84 in eGFR< 45 analysis and OR = 2.03; 95%CI = 1.44–2.87 in eGFR< 30 analysis) also qualified as significant correlates of low EQoL-VAS in model 9.

**Table 4:** Probability of having low quality of life (QoL 0–50) in CKD groups with older adults with eGFR < 45 ml/min/1.73 m^2^ vs. older adults with eGFR > =45 ml/min/1.73 m^2^ (left column), and right column CKD groups with older adults with eGFR < 30 ml/min/1.73 m^2^ vs. eGFR > =30 ml/min/1.73 m^2^

Predictors	OR (95% CI) GFR < 60	OR (95% CI) GFR < 45	OR (95% CI) GFR < 30
**Model 1.** CKD alone	1.47 (1.16–1.85)	1.38 (1.08–1.77)	1.57 (1.01–2.44)
**Model 2.** Model 1 adjusted for age and sex	1.47 (1.15–1.86)	1.40 (1.07–1.82)	1.71 (1.08–2.69)
**Model 3.** Model 2 adjusted for family arrangement (widow)	1.47 (1.15–1.87)	1.40 (1.07–1.82)	1.71 (1.08–2.70)
**Model 4.** Model 2 adjusted for SPPB total score	1.20 (0.93–1.56)	0.87 (0.64–1.18)	0.84 (0.50–1.42)
**Model 5.** Model 2 adjusted for At least 1 fall past 12 months	1.47 (1.16–1.88)	1.39 (1.06–1.82)	1.70 (1.07–2.69)
**Model 6.** Model 2 adjusted for GDS > 5	1.48 (1.15–1.91)	1.42 (1.07–1.88)	1.78 (1.11–2.87)
**Model 7.** Model 2 adjusted for Cumulative Illness Rating Score and number of medications≥5	1.16 (0.90–1.50)	0.86 (0.64–1.16)	1.11 (0.69–1.80)
**Model 8.** Model 2 adjusted for lower Urinary tract symptoms	1.50 (1.18–1.92)	1.47 (1.12–1.92)	1.93 (1.22–3.07)
**Model 9.** Model 2 adjusted for Diabetes, Hypertension, and Chronic obstructive pulmonary disease	1.28 (0.99–1.64)	1.16 (0.88–1.52)	1.47 (0.92–2.34)
**Model 10.** Model 2 adjusted for Stroke, Hip fracture, and Parkinson’s Disease	1.48 (1.16–1.89)	1.42 (1.08–1.87)	1.65 (1.03–2.62)
**Model 11.** Model 2 adjusted for Anemia	1.47 (1.15–1.86)	1.49 (1.0–2.22)	1.51 (1.01–2.25)

**Abbreviations:** CI, confidence interval; OR, odds ratio

## Discussion

The main finding of the present study is the association between CKD and EQoL-VAS among older community-dwelling people free from end-stage renal disease. Interestingly, such association was confirmed with all eGFR thresholds used (namely, stages 3a, 3b and 4). Thus, our results add to the present knowledge by demonstrating that early stages of CKD may significantly affect QoL among older people.

Former studies clearly showed that end-stage renal disease and dialysis are associated with low QoL [15, 16, 40, 41], and few studies reported that even early stages of CKD may significantly affect QoL [17–20]. Our findings are clearly different from that reported in a recent cross-sectional analysis of the Irish Longitudinal Study on Ageing showing that creatinine-based eGFR may contribute little to QoL [42]. However, difference in age of the enrolled populations likely account for this apparent discrepancy. Indeed, only people aged 75 or more were enrolled in the present study, while people enrolled in the Irish study were younger (median age 61 years, interquartile range 55–68) [42]. Thus, in the light of results from the present study and the above evidence, the need of a patient-centered approach including universal outcomes to CKD care among older people [43] could be further suggested.

Main mechanisms linking CKD to QoL among older people are likely linked to the complex profile of older patients with CKD, which is known to be characterized by impaired physical function [7, 8], frailty [9, 10], cognitive impairment [11], vision impairment [12], malnutrition [13], and sarcopenia [14]. The finding that selected variable, such as physical performance, comorbidity and polypharmacy, may significantly affect the relationship between CKD and QoL is in keeping with such interpretation. Former studies showed that reduced renal function may be associated with poorer physical performance in older patients [7], and impaired SPPB contributes to describe the profile of older CKD patients with increased risk of death [44]. Additionally, besides confirming the impact of physical performance on QoL [45], our study also showed that the average difference across QoL groups observed in our study in regards to SPPB score was clearly higher compared to minimum clinically meaningful difference (i.e. 0.5 points) [46]. These findings further sustain the need of developing exercise interventions to improve physical performance among CKD patients to counteract deterioration of QoL [47, 48].

Overall comorbidity (i.e. CIRS score) and selected diagnoses (i.e. diabetes, hypertension and chronic obstructive pulmonary disease), are known to be major determinant of CKD or highly prevalent comorbidities among older patients with CKD [49, 50]. Diabetes, hypertension and chronic obstructive pulmonary disease were also found associated with QoL decline in dialysis patients [51–53], and our findings are consistent with the hypothesis that these comorbidities may negatively affect QoL even among older people with less severe degrees of CKD. On the other hand, diabetic patients maintaining high level of physical activity and exercise were exhibited better QoL [54]. Finally, CIRS was found associated with QoL in community-dwelling older adults [55], as was polypharmacy [56]. Thus, our findings that the addition of these variables to the multivariable models may blunt the association between CKD and QoL further strengthen their role as important correlates of QoL among older people and suggests that the impact of CKD on QoL may be at least partly mediated by risk factors typically observed among older people.

Limitations of the present study deserve to be mentioned. The cross-sectional design does not allow to derive causal relationships between CKD and QoL. However, the ongoing collection of prospective data in the context of the SCOPE study is expected to provide further insight in this topic. Additionally, we enrolled a population of relatively healthy older community-dwelling volunteer, thus prone to volunteer bias, which may reduce generalizability of the present finding to the general older population. Finally, only creatinine-based eGFR was used as a measure of kidney function in our study, and recent evidence suggests that using different biomarkers (e.g. cystatin C) may yield different results [42]. As for strength, we had the opportunity to investigate the association between CKD and QoL after adjusting for several important confounders thanks to the comprehensive assessment carried out during the study visits.

## Conclusions

Our study shows that in older adults self-perceived QoL is multifactorial and influenced by medical, emotional, functional and social conditions. We observed a significant association of CKD stages 3a, 3b and 4 with QoL. Such association was confirmed after adjusting for sociodemographic and clinical factors. Efforts should be made to decrease the negative effects of potentially modifiable factors, such as physical performance, and to better manage comorbidities. Further longitudinal studies are need to clarify whether targeting patients with early stages of CKD may help to prevent QoL decline.

## Data Availability

Data will be available for SCOPE researchers through the project website (www.scopeproject.eu).

## Abbreviations

QoL: Quality of life
CKD: Chronic Kidney Disease
EQ-VAS: EQ-Visual Analogue Scale
Euro-Qol 5D: Euro-Quality of life 5D
eGFR: Glomerular filtration rate
BIS: Berlin Initiative Study eq.
ADL: Basic Activities of Daily
IADL: Instrumental Activities of Daily Living
GDS: Geriatric Depression Scale
CIRS: Cumulative Illness Rating Scale
LUTS: Lower urinary tract symptoms
SPPB: Short Physical Performance Battery
OR: Odds Ratio
